# Modern Treatments and Stem Cell Therapies for Perianal Crohn's Fistulas

**DOI:** 10.1155/2016/1651570

**Published:** 2016-12-07

**Authors:** Alghalya Khalid Sulaiman Al-Maawali, Phuong Nguyen, P. Terry Phang

**Affiliations:** ^1^St Paul's Hospital, Vancouver BC, Canada; ^2^University of British Columbia, Vancouver BC, Canada

## Abstract

Crohn's disease (CD) is a complex disorder with important incidence in North America. Perianal fistulas occur in about 20% of patients with CD and are almost always classified as complex fistulas. Conventional treatment options have shown different success rates, yet there are data indicating that these approaches cannot achieve total cure and may not improve quality of life of these patients. Fibrin glue, fistula plug, topical tacrolimus, local injection of infliximab, and use of hematopoietic stem cells (HSC) and mesenchymal stem cells (MSC) are newly suggested therapies with variable success rates. Here, we aim to review these novel therapies for the treatment of complex fistulizing CD. Although initial results are promising, randomized studies are needed to prove efficacy of these approaches in curing fistulizing perianal CD.

## 1. Introduction

Crohn's disease (CD) is a complex disorder of uncertain etiology characterized by chronic recurrent inflammation of the bowel. The disease incidence in North America ranged from 3.1 to 20.2 cases per 100,000 persons per year in published epidemiological studies [1, 2]. Perianal fistulas occur in about 20% of patients with CD and are almost always classified as complex fistulas [3]. Parks et al. classified fistulas based on their anatomy of origin, route, and external opening into superficial, intersphincteric, transsphincteric, suprasphincteric, or extrasphincteric [4]. The American Gastroenterology Association (AGA) divided fistulas into simple and complex fistulas based on number of external opening, location, and associated complications. Both are useful and common classification methods when referring to CD perianal fistula disease. The ideal outcome from treatment of these fistulas is complete closure with prevention of infection and abscess formation. However, intensive medical and surgical therapy has only success rates ranging from 30 to 80%. In view of incomplete fistula closure, treatment strategies have shifted from cure to reduction of fistula drainage and quality life improvement until more effective therapies become available.

## 2. Conventional and Biological Medical Treatments

Antibiotics, immunosuppressive drugs such as thiopurines, oral tacrolimus, and anti-TNF alpha's role in the management of fistulizing CD have been reported with variable success rates when used as single agents or in combination (see Table 1). Antibiotics use in uncontrolled studies of fistulizing CD report symptom reduction but fail to result in fistula closure [5, 6]. There was no significant difference between antibiotics and placebo in achieving complete fistula closure or/and improvement of fistula in a small sampled, randomized, double blinded, placebo-control study [7]. Effectiveness of thiopurines, including 6-metacaptopirine and azathioprine, studied by Pearson et al., has been investigated in a meta-analysis of 5 controlled trials reporting complete fistula closure or reduction in fistula drainage in 54% of patients [8]. Multiple studies and randomized controlled trials showed that anti-TNF alpha treatments including infliximab, adalimumab, and certolizumab are superior to placebo in induction treatment and maintenance therapy for perianal fistulas in CD [9–14]. However, development of antibodies against these agents has been reported and can result in loss of clinical response [15]. In addition, anti-TNF agents have been associated with opportunistic infections, serum sickness-like reaction, autoimmune disorders, and sepsis [16]. In a randomized control trial, although oral tacrolimus was effective in closure of 50% of CD fistulas, there was no difference in complete closure of all fistulas when compared to placebo [17].

## 3. Surgical Options

Fistulotomy with sphincterotomy is the preferred management for simple fistulas that results in high cure rates without fecal incontinence in non-CD fistulas. In CD fistulas with any degree of diarrhea, seton placement, advancement flaps, and ligation of the intersphincteric fistula tract (LIFT) are surgical options that have higher recurrence rates in an attempt to avoid fistulotomy with sphincterotomy that could result in incontinence. Seton placement for chronic drainage does not cure fistulas but limits recurring perianal sepsis and is the standard surgical option for CD fistulas that is meant to improve quality of life in patients living with chronic disease [18–22]. Advancement flaps have healing rates from 60 to 70% but have increased complications over seton drains [18, 23, 24]. Data regarding effectiveness of the LIFT procedure in CD patients are lacking [25–27]. Best practice guidelines recommend seton placement as the preferred technique to allow continuous drainage [28, 29].

Current combined medical and surgical management is reported to have better outcomes in the treatment of perianal fistulas in CD [30–32]. Yet, these approaches do not achieve cure and fail to sufficiently improve quality of life of these patients such that there is need for new and improved treatments. Fibrin glue, fistula plug, topical tacrolimus, local injection of infliximab, and the use of hematopoietic stem cells (HSCs) and mesenchymal stem cells (MSCs) are newly suggested therapies for these fistulas.

## 4. Fibrin Glue

Fibrin glue is a mixture of fibrinogen, calcium ions, and thrombin that gets injected using a catheter into the fistulas tract and clots within 60 seconds. Preservation of anal sphincter function is a main advantage of this procedure, but early extravasation of the mixture from the fistulous tract and failure of exact identification of all fistula branches result in high recurrence rate. Data on fibrin glue effectiveness in CD is very limited. In a randomized control trial assessing fibrin glue effectiveness in CD patients, clinical remission was reported in 38% of the study group compared to 16% in the control group [33]. Lindsey et al. reported that only two out of six CD patients treated with fibrin glue (33%) reported healing defined as no drainage. Longer term follow-up data for fibrin glue in CD fistula has not been reported [34].

## 5. Fistula Plug

Fistula plug is a cone shaped plug synthesized from lyophilized porcine small intestine mucosa that is threaded through the fistulous tract and fixed in place with a suture. The strategy of plugs is similar to fibrin glue in simplicity of use and avoidance of injury to the anal sphincter muscle but is meant to decrease failure from dislodgement of glue. Chung et al. reported healing rates of 75% at 12 weeks in 51 inflammatory bowel disease (IBD) patients of which 40 were CD patients [18]. However, most failures were reported to be due to early extrusion of the plug within 1 week after placement. In a systematic review published in 2012 by O'Riordan et al., healing rates in non-CD and CD fistulas were reported to be 54.8% and 54.3%, respectively [35].

## 6. Topical Tacrolimus

Use of topical tacrolimus in CD perianal fistulas was suggested from its effectiveness in treatment of immune mediated skin diseases [36] but studies about its usage in CD are very limited. In a recently published systematic review of tacrolimus use in CD [37], only 1 randomized controlled trial (RCT) was found addressing topical tacrolimus use in fistulizing perianal CD. Hart et al. tested 12 CD fistulizing patients and concluded that there was no benefit from topical tacrolimus [38].

## 7. Intralesional Infliximab

Multiple local injections of infliximab have been placed into the fistulous tract, internal opening, and external opening. Two uncontrolled small sampled studies were published with healing rates around 70% [39, 40]. Asteria et al. excluded patients who received previous treatment with infliximab systemically or had rectal or perianal complications like proctitis or abscess [40]. Local injections of infliximab were effective in 72.7% showing reduction in fistula drainage higher or equal to 50% and 36.4% achieving complete cessation of drainage for a minimum of 4 weeks. Alessandroni et al. combined repeated peri-fistular infliximab injection with core-out fistulectomies as a treatment for refractory complex perianal fistulas in CD [41]. Fistula closure at 12 months was observed in 7 out of 8 patients (87.5%) who completed the treatments. These are promising initial data and controlled randomized studies are needed to prove efficacy [41].

## 8. Stem Cell Therapy

Use of HSC and MSC in treatment of fistulizing CD was proposed after the serendipity of discovery using stem cells in treatment of CD in 1993. The first report of stem cells' ability to treat IBD patients was on a 41-year-old female who underwent an autologous HSC transplantation for non-Hodgkin's lymphoma [42]. The patient had also suffered from Crohn's disease since 1970, had partial colectomy in 1985, and developed rectovaginal fistula in 1986. The patient was given a total of 3.07 × 108/kg nucleated viable CD34^+^ HSCs transplanted as treatment for the lymphoma. To everyone's surprise no active Crohn's was present in the first 6 months following the HSC transplant. She was surprisingly asymptomatic and needing no treatment for Crohn's disease. Another case of successful long-term control of Crohn's disease was subsequently reported in a 20-year-old male with non-Hodgkin's lymphoma who also developed a CD perianal fistula. The CD fistula was in stable condition for 7 years following autologous bone marrow transplantation. There was no clinical or laboratory evidence of recurrence of either Crohn's disease or non-Hodgkin's lymphoma in this patient [43].

CD fistulas likely result mainly from a long-term effect of an autoimmune condition. The concept of resetting the exaggerated immune response with a brand new immune system using HSC transplant is strongly supported by observations of patients after undergoing HSC transplantation [44]. Before new HSCs are administered intravenously into the patient, the patient needs to go through preparative treatments that result in ablation of their current immune system. This procedure will rescue the body from the exaggerated autoimmune condition and permit the new hematopoietic precursors to generate a new tolerant T-cell population [45].

From 1995 to 2007, 22 out of 25 IBD patients who underwent autologous HSC transplant for blood and bone marrow cancer were reported to achieve clinical remission over a median follow-up for 20 months, only two of which received ongoing treatment for Crohn's disease. Even though the original studies were not aimed at investigating the effect of autologous HSC transplant on IBD progression, the notion of long-lasting remission from CD and the potential improvement of related fistulizing disease by resetting new self-tolerant lymphocytes through chemotherapy was supported [46].

In 2010, Burt et al. published a long-term follow-up report on HSC therapy for IBD, in which 24 patients with severe anti-TNF refractory Crohn's disease received nonmyeloablative hematopoietic stem cell transplantation. Among these, 19% stayed in remission for 5 years, 57% for 3 years, and 91% for 1 year after the transplantation [47]. In another trial of autologous HSC transplantation in severe CD patients, there was perianal fistulas closure observed in three out of four patients [48].

Although T-cell depletion of the graft will result in self-tolerance, safety with use of HSC transplantation is of significant concern because of the risks of infectious complication due to prolonged lymphopenia [49]. As such, all studies to date only focus on severe refractory Crohn's disease cases in which the risks are out-weighed by potential benefits. Because allogeneic HSC transplantation has high complication and mortality rates, it is not presently recommended treatment for autoimmune diseases [50]. Recently, a phase I/II trial (ClinicalTrials.gov Identifier: NCT01288053) was designed to evaluate the effects of matched sibling nonmyeloablative allogeneic HSC transplantation on patients with refractory Crohn's disease. Unfortunately, this trial has been terminated because there was no participant enrolled and the group had no plan to continue the study.

## 9. Local Mesenchymal Stem Cell (MSC) Therapy in Fistulizing CD

When cultured, a population of bone marrow derived mononuclear cells will adhere to plastic forming colony units of fibroblast morphology [51]. With the ability to differentiate into various lineages such as adipocytes, osteocytes, and chondrocytes, this population is identified as mesenchymal stem cells (also called mesenchymal stromal cells) [52, 53]. MSCs can self-renew and be maintained for prolonged periods in* ex vivo* condition. Moreover, they are known to have potent anti-inflammatory and immunomodulatory capacities [54]. Adipose tissue derived MSCs (ad-MSCs) can also be isolated from liposuction aspirates and have capability of exerting immunosuppressive functions, providing a promising therapy for inflammatory related tissue injury [55]. With 90% similarity in the immunophenotypes, bone marrow derived MSCs (bm-MSCs) and ad-MSCs are alternatively being used for local treatment of IBD to make wound healing more efficient. However, most current experiments use ad-MSCs due to their higher abundance in human adipose tissue and they can be harvested through liposuction with minimal adverse effects [56, 57]. Ad-MSCs' multilineage capacity has been reported in* in vitro* and animal studies using a colitis mice model showing ad-MSCs' ability to inhibit inflammatory and autoimmune responses [55].

In 2003, a case report was published as one of the first clinical treatments documented on cell therapy treating IBD using ad-MSCs [56] (see Table 2). A 33 year-old woman with Crohn's colitis for 11 years developed perianal suppurations and rectovaginal fistula. Her symptoms did not improve with infliximab and seton drainage. After receiving 9 × 10^6^ adipose tissue derived-MSC local injection, her surgery wound closed completely within one week (with minor signs of inflammation) and there was no fecal incontinence or vaginal flatus being observed. The patient remained asymptomatic with no recurrence of a rectovaginal fistula within 3 months. In 2011, Ciccocioppo et al. published results of a clinical trial involving 10 patients with complex perianal fistula and enterocutaneous fistula [58]. Each patient received two to five intrafistula injections with bm-MSCs every 4 weeks. At 12 months after the final injection, complete closure (seven cases) and incomplete closure (three cases) of fistula tracks were evident with appearance of regenerative tissue in parallel with reduction of Crohn's disease. In trials performed by Garcia-Olmo et al., ad-MSC therapy for complex perianal fistula had significant efficacy (more than 70%) versus fibrin glue in both Crohn's and non-Crohn's patients [59–61].

Recently, phase III ADMIRE trial was conducted on 212 patients with Crohn's disease to assess the safety and efficacy of allogeneic ad-MSCs for treatment of complex perianal fistulas compared to placebo over a 24-week period and extended follow-up period up to 104 weeks (“Adipose derived MSCs for induction of remission in perianal fistulizing Crohn's disease”; ClinicalTrials.gov Identifier: NCT01541579). Patients with complex perianal fistulas were randomly assigned to single intralesional injection of 120 × 10^6^ allogeneic, expanded ad-MSCs (Cx601, 107 cases), or placebo (105 cases). At week 24, it was found that a significantly higher percentage of patients treated with Cx601 versus placebo achieved combined remission (50% versus 34%). Only 17% of patients in the Cx601 group (compared to 29% in the placebo group) experienced treatment-related adverse events. These preliminary results indicate that Cx601 is more effective and safer to treat complex perianal fistulas in patients with Crohn's disease [62]. Another phase I study of autologous mesenchymal stromal cell coated fistula plug in patients with fistulizing Crohn's disease is currently recruiting participants (“Stem cell fistula plug in perianal Crohn's disease”; ClinicalTrials.gov Identifier: NCT01915927). The primary endpoint of this study is to determine the safety and feasibility of using ad-autologous MSC coated Gore Bio-A Fistula Plug as an option for new treatment.

With the growing interest in MSCs therapy for fistulizing Crohn's disease, researchers still need to overcome many existing obstacles in the design of their studies. Of note, a number of clinical trials have been terminated because they did not provide a feasible uniform protocol in cell isolation and selection, and cell expansion technique was not fully developed. Between different existing trials, MSCs dosage needed for optimized results has yet to be determined. Up to date, there are only a handful number of completed clinical trials performed mostly on a small group of patients with n value ranging from 3 to 24, and successful rates vary from case to case [63]. The majority of these studies only involved patients who have failed and discontinued standard therapies before being enrolled into the trials. In order to have a better knowledge on stem cell therapy, experiments involving larger number of patients are still ongoing with earliest estimated completion date in 2017 [62], and more studies are being designed to include patients treated with MSCs in combination with biological drugs to investigate the possibly magnified effects brought by two methods or any complexity they might bring [64]. It is also suggested that, for higher clinical efficacy, robust priming for best MSCs culture needs to be done in advance together with a careful donor selection process and identification/isolation of its subpopulation with enhanced immunosuppressive properties [49]. Furthermore, we do not yet have a good experiment set-up* in vivo* showing the multilineage ability of MSCs and how to prevent MSCs from turning into something undesirable within patients. Obviously, there are still a lot of questions to be answered before MSCs can be fully accepted as a novel and safe therapy for fistulizing Crohn's disease.

## 10. Conclusion

Treatment of CD fistulas remains challenging with persisting detrimental effect on quality of life in patients living with chronic disease. New local treatments including fistula plugs and local injection of infliximab after abscess drainage report promising early results but randomized trials of larger patient numbers and longer term follow-up are required. A handful of clinical studies using stem cell therapy have shown positive results where it is found to be safe and effective for patients who did not respond to biological treatments. However, HSC and MSC therapies are not yet fully developed for technical procedure to be routinely and safely performed. There remains a therapeutic gap between conventional treatments and cure of fistulizing CD until clinical trials prove efficacy of these novel treatments.

## Figures and Tables

**Table 1:** Summary of conventional and biological medical treatments of fistulizing CD and reported outcomes.

Treatment	Studies	Outcome
Antibiotics	Brandt et al. [5], Prantera et al. [6]; uncontrolled studies	Symptoms reduction but failed closure
	Thia et al. [7] an RCT; AB versus placebo.	No significant difference
Thiopurines	Pearson et al. [8] a meta-analysis; Thio versus placebo	Complete fistula closure or reduction in fistula drainage in 54% of patients
Anti-TNF alpha		
Infliximab	Present study [9] 5 mg/kg versus 10 mg/kg versus placebo	≥50% closure; 68% versus 56% versus 26% (*p* = 0.002, *p* = 0.02, resp.)
	ACCENT II study [10] infliximab versus placebo	Maintenance of complete closure of draining fistula; 36% versus 19% (*p* = 0.009)
Adalimumab	CHARM study [12] adalimumab versus placebo	Complete fistula healing at 56 wks; 33% versus 13% (*p* < 0.05)
	ADHERE study [13]	23% fistula remission, 41% fistula improvement
Certolizumab pegol	Schreiber et al. [14] an RCT; certolizumab pegol versus placebo	Complete closure at 26 weeks; 36% versus 17% (*p* = 0.038)

**Table 2:** Published and ongoing clinical trials using MSCs for the treatment of Crohn's perianal fistula.

Authors/trial code	Study design	Status and results
García-Olmo et al. 2005 [59]	*n* = 4 (phase I study) Autologous adipose derived stem cells	Trial completed 75% of fistulas have complete closure at week 8
Garcia-Olmo et al. 2009 [60]	*n* = 14 (phase II study) Autologous adipose derived stem cells + fibrin glue	Trial completed Fistula healing: 71% versus 16%
Ciccocioppo et al. 2011 [58]	*n* = 10 (prospective study) Autologous mesenchymal stem cells	Reduction in Crohn's disease activity index and pain/discharge scores
Herreros et al. 2012 [61] NCT00475410	*n* = 200 (phase III study) Autologous adipose derived stem cells + fibrin glue	Trial completed At 1 year, the healing rates were 57.1% (stem cells alone), 52.4% (stem cells + fibrin glue), and 37.3% (fibrin glue alone)
Molendijk et al. 2015 [64] NCT01144962	*n* = 21 (phase II study) Allogeneic adipose derived stem cells Placebo-control	Trial completed Healing up to 85%
Panés et al. 2016 [62] NCT01541579	*n* = 212 (Phase III study) Allogeneic adipose derived stem cells versus placebo	Trial ongoing 50% versus 34% achieved combined remission
NCT01915927	(Phase I study) Autologous adipose derived stem cells coated fistula plug	Trial recruiting patients
